# Lettuce Contamination and Survival of *Salmonella* Typhimurium and *Listeria monocytogenes* in Hydroponic Nutrient Film Technique Systems

**DOI:** 10.3390/foods11213508

**Published:** 2022-11-04

**Authors:** Sanja Ilic, Margaret R. Moodispaw, Lawrence V. Madden, Melanie L. Lewis Ivey

**Affiliations:** 1 Human Nutrition, Department of Human Sciences, College of Education and Human Ecology, The Ohio State University, Columbus, OH 43210, USA; 2 Department of Plant Pathology, College of Food, Agricultural and Environmental Sciences-Wooster, The Ohio State University, Wooster, OH 44691, USA

**Keywords:** food safety, hydroponics, nutrient film technique, *Salmonella*, *Listeria monocytogenes*, water quality, prevention, nutrient

## Abstract

Hydroponic vegetable production is increasing globally, but there is a lack of science-based recommendations to ensure their food safety. Specifically, there is limited evidence for establishing water management strategies. The purpose of this study was to determine the survival of *Salmonella* Typhimurium and *Listeria monocytogenes* in commercial nutrient flow technology (NFT) systems during the lifecycle of lettuce exposed to sporadic or extreme contamination. NFT systems were inoculated with *Salmonella* Typhimurium or *Listeria monocytogenes*, and nutrient solution, rockwool, roots, and lettuce leaves were collected over the lettuce production cycle for pathogen enumeration and detection. Both human pathogens persisted in the lettuce NFT growing system throughout the growth cycle of lettuce. *Salmonella* Typhimurium and *L. monocytogenes* accumulated in rockwool medium and on lettuce roots and were transferred to the leaves at quantifiable levels from the contaminated nutrient solution. In the nutrient solution, *Salmonella* concentration under sporadic and extreme conditions declined significantly 24 h after inoculation and again 7 days post-inoculation (*p* < 0.0001). Under extreme conditions, the concentration did not change significantly after 7 days, while under sporadic conditions, the concentration declined again 14 days post-inoculation in the nutrient solution collected from the reservoirs. *L. monocytogenes* populations in the nutrient solution fluctuated significantly over the 28-day growth cycle (*p* < 0.0001). Under extreme conditions, *L. monocytogenes* concentrations in the nutrient solution declined, while under sporadic conditions, the populations increased. The findings of this study, for the first time, describe human pathogen survival in commerical NFT systems and highlight the urgent need for novel approaches to mitigating the risks from nutrient solution contaminaiton in hydroponics.

## 1. Introduction

The production of fruits and vegetables in controlled environments is on the rise in the United States (US) and globally due to the increased consumer demand for out-of-season fresh produce. Approximately 75% of fresh-market tomatoes, 30% of peppers and cucumbers, and an increasing market share of leafy greens (lettuce and leafy herbs) sold in the US are produced in controlled environments [1], with a projected growth of 188.4% by 2026 [2]. Hydroponic farms have higher crop yield per acreage and other several advantages, including the reduction in water waste [3,4,5,6] and chemical use (i.e., pesticides) and the ability to grow produce in climate-change-affected areas [7,8]. However, controlled environmental conditions (e.g., temperature, humidity, etc.) in hydroponic greenhouses that allow for intensive year-round production are also highly conducive for the survival, transfer, and growth of foodborne pathogens such as *Listeria monocytogenes* and *Salmonella* spp. that can cause severe infections.

The potential for the contamination of greenhouse-grown produce is well documented [9,10,11,12]. In addition to the isolation of *Salmonella* Saintpaul from puddles inside a greenhouse [13,14], *L. monocytogenes* was isolated from contact and non-contact surfaces in commercial tomato greenhouses in the US [15]. Not surprisingly, an increasing number of foodborne illness outbreaks have been linked to produce grown hydroponically. In 2011, 4321 people became ill, and 22% developed hemolytic-uremic syndrome (HUS) in a large *Escherichia coli* O104:H4 outbreak in Germany from contaminated fenugreek sprouts produced in hydroponic cultures, and the same strain was isolated from hydroponic cucumbers during the outbreak investigation [10], illustrating the extent of the risk of contamination in different hydroponic crops. In 2013, 84 people in 18 states were infected by *Salmonella* Saintpaul by consuming greenhouse cucumbers [11]. Since this time, multiple outbreaks due to hydroponically grown sprouts have been recorded [16], and most recently, a *Salmonella* Typhimurium outbreak from packaged hydroponic salad greens sickened 31 people and hospitalized four in four states in the US [17].

Food safety is critical for consumers’ health and well-being [18,19]. While leafy greens are estimated to be the most common cause of foodborne illness in the US [20], they are also essential for human nutrition. Because of this, it is imperative that any contamination with human pathogens is prevented. However, because current food safety research has focused on field production, the hydroponic industry is lacking validated strategies to prevent contamination effectively. The current standards for the prevention of leafy green contamination are based on the US Food and Drug Administration (FDA) Food Safety Modernization Act (FSMA) requirements and its Produce Safety Rule (US Code of Federal Regulations (CFR) [21]), developed for field production. The implementation of food safety measures is not readily transferable from the field to hydroponic production, making it difficult for the hydroponic industry to achieve these requirements. This is particularly true for production water/nutrient solutions.

Most hydroponic leafy greens are produced in nutrient film technology (NFT) systems, where a thin film of nutrient solution re-circulates from the reservoir through multiple channels and flows directly over the crop roots. This system uses recirculated water to deliver nutrients and pesticides to the plants, and water has been shown to readily transfer pathogens to the roots, where they can colonize the root system systemically [22,23]. Further, in vitro studies found that once *Salmonella* spp. establishes on surfaces in hydroponic systems (such as the inner sides of the reservoirs and channels), it could persist for extended periods of time under typical conditions that are maintained in greenhouses (temperature 24–29 °C, relative humidity 80–90%) and cross-contaminate produce downstream [14,24]. The risks of crop contamination in commercial hydroponic systems are not well defined, and the current research lacks answers regarding the survival and persistence of human pathogens in contaminated hydroponic systems during crop production and regarding how to mitigate contamination [25]. In addition, the risks of contamination can vary depending on how the system is set up and the management practices—specifically, the location of the reservoir and the climatic conditions [25].

Only limited data are available to develop good agricultural practice guidelines for the hydroponic industry and effectively prevent foodborne infections linked to hydroponically grown produce. The objectives of this study were to assess the contamination risks of lettuce grown in commercial NFT systems from a contaminated nutrient solution by quantifying the survival of *S. typhimurium* and *L*. *monocytogenes* in the nutrient solution and their transfer to lettuce roots and leaves under sporadic and extreme contamination events.

## 2. Materials and Methods

**Figure 1:**
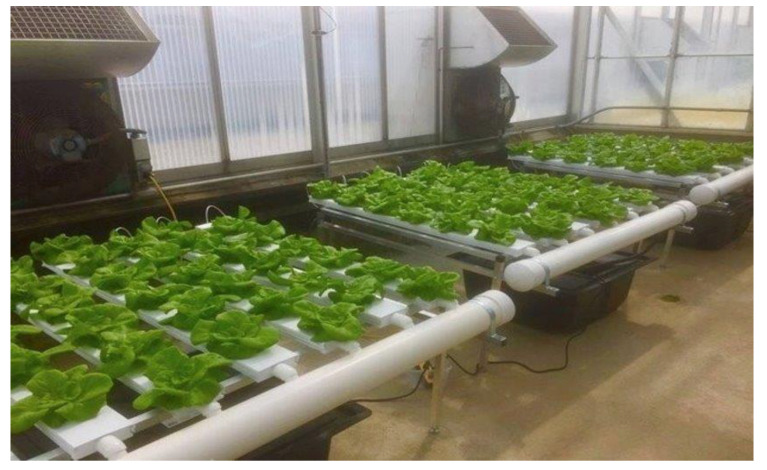
Nutrient Film Technology (NFT) system units with four channels containing six plant spaces (NFT 4–6) fitted with 25-gal (95 lit) reservoir and the pump for re-circulating nutrient solution (CropKing Inc., Lodi, OH, USA) set up in Biosafety Level 2 (BSL-2) greenhouse space at the Ohio State University (Wooster, OH, USA).

Nutrient flow technique (NFT) set up. Small-scale NFT 4–6 hydroponic systems (CropKing Inc., Lodi, OH, USA) were used in this study. Each system contained six 1.2 m food-grade PVC channels with removable covers, and each channel held six plants for a total of 36 plants per system (Figure 1).

Nutrients were held in 25 gal (95 lit) reservoirs made from ABS plastic with UV inhibitors, recirculated using a 1514 LPH submersible pump (Active Aqua, New Hudson, MI, USA), and aerated with an ImagitariumTM air pump (Petco, San Diego, CA, USA). Each reservoir was equipped with a Bluelab Guardian Monitor (Bluelab Corporation Limited, Tauranga, New Zealand) for measuring pH, electrical conductivity (EC), and temperature (°C).

Seedling and plant production. Lettuce cv. Rex seeds were sown into 200-cube rockwool pads (Grodan, Roermond, Netherlands) saturated with a commercial formulation of a nutrient solution supplemented with calcium nitrate (Hydro-Grow Leafy Greens, CropKing Inc., Lodi, OH, USA), according to the manufacturer’s recommendation. The seedlings were grown to the 3-to-4 true leaf stage (~2 weeks) before being transplanted into the NFT systems containing the Hydro-Grow Leafy Greens nutrient solution (pH 5.5 to 6.0). Seedlings and plants were produced in a biosafety level 2 greenhouse located at CFAES—Wooster (Wooster, OH, USA). Greenhouse temperatures ranged from 12 to 30 °C, depending on the season. Fifty percent shade cloth (Aluminet, Alion Home Inc., Bassett, CA, USA) was used from June through August to reduce light intensity and temperature.

Bacterial strains and nutrient solution inoculation. Salmonella enterica subsp. *Enterica ^ser^*Typhimurium LT2 strain JSG626 responsible for gastroenteritis in humans (Deblais et al. [26] or the *L. monocytogenes* strain Pirie (ATCC 19111) were used to contaminate the nutrient solution. To recover *L. monocytogenes* and *Salmonella* Typhimurium from LB:glycerol (*v*:*v* 1:1) stock at −80 °C, the strains were plated onto LB agar amended with 100 µg/mL nalidixic acid (LB^na^) or xylose lysine deoxycholate (XLD) amended with 75 µg/mL kanamycin (XLD^ka^), respectively. The cultures were incubated at 35–37 °C for 48 h before adding a single colony to 10 mL LB broth. The inoculum was grown in 2 L flasks and incubated with shaking at 120 rpm for another 16 h. The liquid cultures were then pelleted by centrifugation (4122× *g*, 5 min), and the pellets were washed twice with 1X phosphate buffered saline (PBS) (pH 7.4, 0.01 M) and suspended in nutrient solution. The seedlings were transplanted and allowed two days to acclimatize in the system. The nutrient solution in the reservoirs was then inoculated to simulate a sporadic (~10^4^ CFU/mL) and an extreme (~10^7^ CFU/mL) contamination scenario. Upon inoculation, the nutrient solution in the reservoir was homogenized by stirring for 2 min, and the first set of samples was collected at 1 h post-inoculation.

**Figure 2:**
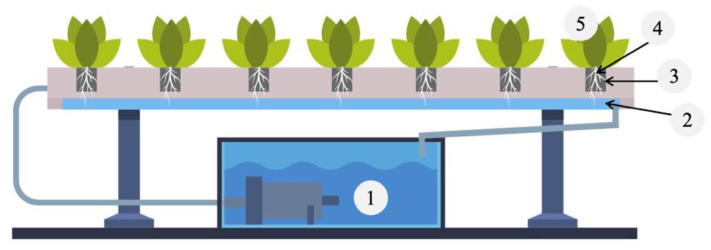
Samples (N = 200) were collected from the nutrient flow technology (NFT) system at 1-, 12-, and 24-h, and 7-, 14-, 21- and 28-days post-inoculation with Salmonella enterica serotype Typhimurium (LT2 JSG626) or Listeria monocytogenes (ATCC 19111). Five replicate samples of nutrient solution from the reservoirs (1) and channels (2), and from rockwool (3), roots (4), and shoot tissue (5) were collected.

Sampling and bacterial enumeration. The nutrient solution from the reservoirs and the channels, roots, rockwool, and leaf tissue (Figure 2) were sampled 1, 12, and 24 h and 7, 14, 21, and 28 days post-inoculation. At each sampling time, five replicate samples were collected. The nutrient solution from the reservoirs (100 mL) was collected by submerging a sterile sample bottle (Nalge Nunc International, Rochester, NY, USA) into the solution and from the channels (1.7 mL) using a pipette.

The pH, EC, and temperature of the nutrient solution were monitored three times daily and recorded at each sampling time. Five plants were randomly selected, and the leaf tissue was separated from the root/rockwool matrix. The leaves, roots, and rockwool samples were placed into 750 mL Whirl-Pak bags (MiliporeSigma, St. Louis, MO, USA) and weighed. Ten mL of 1X PBS was added to all samples other than rockwool, and bacteria were dislodged from the samples using the masticator IUL Masticator Basic (400 mL) Analog Lab Blender (ThermoFisher Scientific, Waltham, MA, USA) for 2 min and a pulsifier (Microbiology International, Frederick, MD, USA) for 1 min. The nutrient solution samples from the reservoir were vigorously shaken by hand for 1 min while the nutrient solutions from the channels were vortexed for 10 secs. All the samples were then 10-fold serially diluted and plated onto LB^na^ for *L. monocytogenes* or XLD^kan^ for *Salmonella* Typhimurium. The cultures were incubated for 24–48 h at 35 °C, and the colonies were enumerated. The cell concentrations (CFU/g of the leaves, roots, or rockwool and CFU/mL of the reservoir and channel nutrient solution) were calculated at each sampling time. The samples were enriched with universal pre-enrichment Broth (UPB, Neogen, Lansing, MI) or tetrathionate bile broth (TBB, Oxoid, Hampshire, UK) for *L. monocytogenes* and *Salmonella* Typhimurium, respectively. The enriched samples were incubated at 35 °C with shaking (175 rpm) for 24 h and then spread plated (0.1 mL) onto XLD^ka^ or LB^na^, and incubated for an additional 24 h at 35 °C. The presence or absence of *Salmonella* Typhimurium or *L. monocytogenes* was determined based on colony morphology on the corresponding medium after enrichment. The experiments representing sporadic contamination were conducted three times, while the experiments representing an extreme contamination event were conducted twice.

Data analysis. The effects of post-inoculation time on the density of the *Salmonella enterica* serotype Typhimurium (LT2 JSG626) and *L. monocytogenes* (ATCC 19111) were analyzed using linear mixed models [27]. The study (i.e., experiment repetition) was considered a random effect, and time was considered a fixed-effect repeated measure. A compound symmetry error correlation structure was specified for the time effect [27]. The response variable was log(CFU+1). Separate analyses were performed for the two pathogens—extreme or sporadic contamination—and for the reservoir nutrient solution, channel nutrient solution, roots, rockwool, and edible leaf tissue. The results were combined across studies for each analysis because the study was not significant (*p* > 0.2). Least squares means were calculated for each time, and the pairwise contrasts of these means were calculated to determine the significant differences between time points (at *p* = 0.05). The data were analyzed in SAS Analytical Software (Cary, NC, USA: SAS Institute Inc.)

## 3. Results

### 3.1. Survival of Salmonella Typhimurium in NFT Hydroponic Systems under Extreme and Sporadic Contamination Events

The *Salmonella* Typhimurium populations in the NFT reservoir and the channel nutrient solution persisted for 28 days post-inoculation under sporadic and extreme contamination conditions. Under both conditions, the percent reduction of bacteria in the nutrient solution (reservoir and channels) was 90% after 24 h, and by the end of the production cycle (28 days), it declined to less than 1 log CFU/mL but remained detectable regardless of the initial inoculation level. Under extreme conditions (Table 1), by day 7, the LS-mean populations in the nutrient solution significantly decreased to 1.18 and 1.19 in the reservoir and channels, respectively (*p* < 0.0001), after which the counts remained similar until harvest. Under sporadic conditions (Table 2), the LS-mean *Salmonella* counts further declined in the nutrient solution between 24 h and 7 days (*p* < 0.0001), after which it was detectable throughout the 28-day production cycle.

Under both conditions, within the first hour post inoculation, the LS-mean counts on the roots were 6.21 (extreme) and 5.72 (sporadic) log CFU/g (Table 1 and Table 2). The populations declined significantly at 24 h post-inculcation under extreme conditions (*p* < 0.0001). Under sporadic conditions, the LS-mean populations did not decline significantly until 7 days post-inoculation, at which point the LS-Mean was 4.34 CFU/g. After 14 days post-inoculation, there was no significant decrease in *Salmonella* counts on the roots under sporadic or extreme conditions, and over 2 log CFU/g were persisting on the roots by harvest (28 days).

In the rockwool, a significant decline in *Salmonella* Typhimurium did not occur until day 7 under extreme conditions and until day 14 under sporadic conditions (Table 1 and Table 2). Like the roots, the *Salmonella* Typhimurium counts at the day of harvest were around 2 log CFU/g.

*Salmonella* Typhimurium transferred to the edible portions of the crop from the nutrients solutions and could be enumerated immediately (1 h) from the leaves under extreme conditions and by 12 h post-inoculation under sporadic conditions (Table 1 and Table 2). Although *Salmonella* Typhimurium could not be enumerated by the culturing techniques in this study at 28 days post-inoculation under extreme conditions and at 1 and 24 h under sporadic conditions, it was detected after enrichment.

### 3.2. Survival of Listeria monocytogenes in NFT Systems under Extreme and Sporadic Contamination Events

*Listeria monocytogenes* populations in the NFT reservoir and channel nutrient solution persisted for 28 days post-inoculation under sporadic and extreme contamination conditions. Over the first 24 h, the LS-mean population decreased significantly in the nutrient solution within the reservoir (*p* = 0.0169) and channels (*p* = 0.0312) (Table 3). While the mean population fluctuated between days 7 and 28, there were no significant differences in *L. monocytogenes* counts within the nutrient solution in the reservoir or channels at harvest. In fact, in the channel, the LS-mean population was similar in the nutrient solution at harvest (day 28) and 1 h post-inoculation.

Under sporadic conditions (Table 4), a significant increase in the LS-mean population was observed on day 7 post-inoculation in both the reservoir and channel nutrient solutions (*p* < 0.0001). At the day of harvest (28 days post-inoculation), the pathogen counts in the nutrient solutions (reservoir and channel) were significantly higher than they were 12 h post-inoculation.

The *Listeria monocytogenes* counts in the roots under extreme conditions were initially high and then significantly decreased on day 7 (*p* = 0.0047; Table 3). The LS-mean populations then increased on day 28 to the initial counts (1 h post-inoculation). Under sporadic conditions, the LS-mean population of *L. monocytogenes* in the roots did not significantly change during the 28-day production cycle and were consistently high (log 5 CFU/g) at all time points (*p* = 0.2913).

Under both conditions, the LS-mean populations of *L. monocytogenes* in the rockwool were high (Table 3 and Table 4). Under extreme conditions, only slight changes in the concentration were observed (*p* = 0.0826). Under sporadic conditions, the counts significantly fluctuated (*p* <0.0001) but were similar on days 1 and 28 post-inoculation.

*Listeria monocytogenes* transferred to the edible portions of the crop from the nutrient solutions and could be enumerated 12 h post-inoculation under extreme conditions and 1 h post-inoculation under sporadic conditions (Table 3 and Table 4). Although *L. monocytogenes* was not enumerated on the leaves at 1 h post-inoculation under extreme conditions, it was detected after enrichment (Table 3). The LS-mean populations on the leaves increased significantly at 24 h post-inoculation under extreme conditions (*p* = 0.0004). Under sporadic conditions, the counts decreased significantly at 12 h post-inoculation (*p* = 0.0135) and then increased again and were similar to the initial concentrations at 28 days (1 h post-inoculation) (Table 4).

## 4. Discussion

In this study, we assessed and described the contamination risks in lettuce grown in commercial NFT systems from a contaminated nutrient solution. For the first time, we quantified the survival of *S.* Typhimurium and *L*. *monocytogenes* in a nutrient solution, roots, and a rockwool medium in the system with crops and the transfer of the pathogens to the edible parts of lettuce. We showed that if *Salmonella* Typhimurium or *L. monocytogenes* ingress into the greenhouse and contaminate the nutrient solution either through sporadic contamination or during an extreme event, such as flooding or storm damage, both pathogens will survive in the system and will be detectible at harvest time, illustrating the importance of food safety preventive measures in hydroponics. Our findings are expected since human bacterial pathogens have previously been shown to survive well in liquid environments. Shaw et al. [24] showed the survival and growth of STEC and *Salmonella* spp. in water and a nutrient solution under laboratory conditions. While we did not observe the growth of *Salmonella* Typhimurium in the system, it was detectible at all sampling points. Xylia et al. [28], examined the effects pH and temperature on *Salmonella* spp. survival in a hydroponic environment and found that the pathogen survived at the pH and temperature ranges commonly used in hydroponics [28], highlighting the conducive conditions for bacterial pathogens. The survival of *L. monocytogenes* in a nutrient solution in a hydroponic system has, for the first time, been shown in our study. This, in conjunction with the previous evidence on *L. monocytogenes* establishment in similar production environments [25], highlights the food safety risks in hydroponics that are often overlooked.

In addition to the ability of the tested human pathogens to survive in the system throughout the lettuce growth cycle, we showed that both pathogens, if present in the nutrient solution, will readily transfer to the edible lettuce leaves. Pre-harvest agricultural water has been a known source of contamination in multiple recalls and foodborne illness outbreaks linked to produce grown in the field [21,29,30,31]. It has been previously shown that water can readily cross-contaminate edible portions of fresh produce during the production and harvest. In fact, cross-contamination via irrigation water [30,32,33,34] and production water [30,32,35] has been shown and recognized as an important route of fresh produce contamination with human pathogens. The FSMA Produce Safety Rule has water quality standards that require the grower to test and monitor the irrigation water [21]. However, the standards are developed for the field conditions and the water sources that are commonly used in open fields, in which the plants are exposed to an open environment and UV-radiation, and, in general, have much lower surface area contact between the plant and irrigation water for a much shorter contact period. In addition, the FSMA water standard pertains only to water that directly contacts the edible part of produce. However, we show that, despite the absence of direct contact with edible leaves in NFTs, where the thin layer of the nutrient film contacts only roots, the risk of human pathogen contamination via a nutrient solution is extremely high. In fact, if the pathogen is present in the nutrient solution, transmission is imminent due to cross-contamination via splash or close contact. This has important implications on future recommendations for water management and should be considered as the FDA revises the irrigation water standards. For example, pathogen die-off rates over time must not be used because, in the control environment, the pathogens persist over time, regardless of the initial contamination level (sporadic vs. extreme). Further, the lack of understanding about leafy green contamination via a contaminated nutrient solution in hydroponic production has previously been identified among growers [36], and the research community has yet to reach a consensus on how to approach potential contamination events in hydroponic farms [25]. Thus, our study filled a critical knowledge gap by providing important evidence about the behavior of bacterial human pathogens and the extent of food safety risks due to a contaminated nutrient solution in hydroponic lettuce, which is critical to the design of any effective food safety management practice for hydroponic water.

In our study, both *Salmonella* Typhimurium and *L. monocytogenes* concentrated in lettuce roots and rockwool cubes. This is supported by the previous findings of Warriner et al. [37], where the interaction of *E. coli* and the roots of hydroponic spinach plants was studied. Although not a human pathogen, a study of bacterial biocontrol against plant diseases on cucumbers also found that *Paenibacillus polymyxa* was able to colonize the roots and rockwool [38], thus illustrating that bacteria can accumulate in rockwool and roots. This potential for accumulation is important, as it can directly affect consumer health, especially when the lettuce heads are sold as “living lettuce” with the roots and rockwool attached. For the growers and regulators, this has important implications, because the concentration of contaminants in a nutrient solution will decrease during the growth cycle due to pathogen migration and accumulation in the root/rockwool matrix, making it more difficult to detect the pathogen by testing the nutrient solution alone. This highlights the importance of the targeted approaches to water quality assessment in hydroponics by the growers when establishing food safety plans, as well as by the regulators when they propose agricultural water standards.

## 5. Conclusions

*Salmonella* Typhimurium and *Listeria monocytogenes* survive well in NFT systems under sporadic and extreme contamination events during lettuce production until harvest and can readily cross-contaminate edible leaves of the leafy greens. Furthermore, both *Salmonella* Typhimurium and *L. monocytogenes* accumulate in the roots and rockwool at high concentrations. These findings impact growers and consumers. A contamination event will lead to crop loss for the grower and, if undetected, can lead to an outbreak, causing illness to consumers. The information gained from this study highlights the need for further research into novel interventions to mitigate these risks.

## Figures and Tables

**Table 1:** Survival of *Salmonella enterica serotype* Typhimurium (LT2 JSG626) following the inoculation of the nutrient solution, simulating an extreme contamination event, in the reservoir and the channels, root, rockwool, and edible leaf tissue over a 28-day production cycle.

Time Post-Inoculation	Reservoir Nutrient Solution ^1^	Channel Nutrient Solution	Root	Rockwool	Edible Leaf Tissue
1 h	4.29 a ^2^	3.91 a	6.21 a	3.97 a	0.97
12 h	3.07 b	2.67 b	5.34 b	3.66 ab	0.08
24 h	2.99 b	2.96 b	3.95 c	3.30 abc	1.02
7 days	1.18 c	1.19 c	3.70 cd	3.16 bc	0.39
14 days	0.56 c	0.79 c	2.96 de	2.19 d	0.55
21 days	0.52 c	0.65 c	3.03 de	2.76 cd	0.30
28 days	0.60 c	0.80 c	2.90 e	2.22 d	0.00 ^3^
*p* value	<0.0001	<0.0001	<0.0001	<0.0001	0.2151

^1^ Each data point represents the combined LS-means (log CFU/mL or CFU/g) of two independent studies. ^2^ Values in a column followed by different letters are significantly different at *p* < 0.05. ^3^ The pathogen detected after enrichment.

**Table 2:** Survival of *Salmonella enterica* serotype Typhimurium (LT2 JSG626) following the inoculation of the nutrient solution, simulating a sporadic contamination event, in the reservoir and the channels, root, rockwool, and edible leaf tissue over a 28-day growth cycle.

Time Post-Inoculation	Reservoir Nutrient Solution ^1^	Channel Nutrient Solution	Root	Rockwool	Edible Leaf Tissue
1 h	2.21 a ^2^	2.19 ab	5.72 a	3.70 ab	0.00 ^3^
12 h	1.54 b	1.99 b	6.24 a	3.78 ab	0.36
24 h	2.22 a	2.30 a	5.55 a	4.26 a	0.00 ^3^
7 days	0.93 c	0.54 c	4.34 b	3.28 b	0.43
14 days	0.44 d	0.65 c	3.35 c	2.52 c	0.14
21 days	0.38 d	0.53 c	2.10 d	2.18 cd	0.11
28 days	0.30 d	0.28 d	2.11 d	1.81 d	0.23
*p* value	<0.0001	<0.0001	<0.0001	<0.0001	0.5171

^1^ Each data point represents the combined LS-means (log CFU/mL or CFU/g) of three independent studies. ^2^ Values in a column followed by different letters are significantly different at *p* < 0.05. ^3^ The pathogen detected after enrichment.

**Table 3:** Survival of *Listeria monocytogenes* (ATCC 19111) following the inoculation of the nutrient solution, simulating an extreme event, in the reservoir and the channels, root, rockwool, and edible leaf tissue over a 28-day growth cycle.

Time Post-Inoculation	Reservoir Nutrient Solution ^1^	Channel Nutrient Solution	Root	Rockwool	Edible Leaf Tissue
1 h	2.49 a ^2^	2.57 a	5.33 ab	4.35	0.00 c ^3^
12 h	1.64 ab	1.85 ab	5.65 ab	4.21	0.19 bc
24 h	1.18 bc	1.33 bc	5.27 ab	3.46	1.12 ab
7 days	0.90 bc	0.79 bc	3.79 c	3.01	1.99 a
14 days	0.84 bc	1.31 bc	5.52 ab	4.05	1.47 a
21 days	0.57 c	0.59 c	4.82 bc	4.28	1.29 a
28 days	1.24 bc	1.52 abc	6.07 a	3.91	1.74 a
*p* value	0.0169	0.0312	0.0047	0.0826	0.0004

^1^ Each data point represents the combined LS-means (log CFU/mL or CFU/g) of two independent studies. ^2^ Values in a column followed by different letters are significantly different at *p* < 0.05. ^3^ The pathogen detected after enrichment.

**Table 4:** Survival of *Listeria monocytogenes* (ATCC 19111) following the inoculation of the nutrient solution, simulating a sporadic contamination event, in the reservoir and the channels, root, rockwool, and edible leaf tissue over a 28-day growth cycle.

Time Post-Inoculation	Reservoir Nutrient Solution ^1^	Channel Nutrient Solution	Root	Rockwool	Edible Leaf Tissue
1 h	- ^2^	- ^2^	5.43	3.49 abc	1.93 ab
12 h	1.35 b ^3^	1.22 c	5.53	2.73 d	0.84 c
24 h	1.13 b	1.35 c	5.76	3.13 cd	2.21 ab
7 days	1.87 a	2.22 a	5.73	3.72 ab	2.39 a
14 days	1.90 a	1.89 ab	5.30	3.47 bc	1.85 ab
21 days	1.34 b	1.60 bc	5.06	3.91 ab	1.53 bc
28 days	1.85 a	2.12 a	5.38	3.97 a	1.82 ab
*p* value	<0.0001	<0.0001	0.2913	<0.0001	0.0135

^1^ Each data point represents the combined LS-means (log CFU/mL or CFU/g) of three independent studies. ^2^ The measures not taken. ^3^ Values in a column followed by different letters are significantly different at *p* < 0.05.

## Data Availability

The data presented in this study are available on request from the corresponding author.
